# CHARACTERIZING CONCURRENT ALZHEIMER’S DISEASE AND CANCER IN U.S. ADULTS OVER 65

**DOI:** 10.1093/geroni/igz038.1725

**Published:** 2019-11-08

**Authors:** Melody K Schiaffino, James Murphy

**Affiliations:** 1 SDSU, San Diego, California, United States; 2 UCSD Moores Cancer Center, La Jolla, California, United States

## Abstract

Cancer (CA) care delivery fragmentation persists for patients across the cancer continuum. Racial and ethnic disparities are one of the primary factors attributable for variation in treatment outcomes, in addition to language and patient-provider communication barriers. Latino and African-American communities also bear a greater burden of Alzheimer’s Disease (AD) risk than White making patients experiencing AD+CA at risk for poor quality and treatment disparities. This study aims to characterize AD+CA in a population-based sample. Using 2004-2013 SEER-Medicare data we identified multiple cancers and the prevalence of concurrent AD+CA in the database (N=273,349). Patients selected for a first primary, histologically confirmed, any stage, not diagnosed in death certificate or autopsy and had at least 24 months of data prior to diagnosis to calculate a comorbidity index. All analyses were conducted in SAS 9.4 (Cary, N.C.). Across lung (LC), colorectal, head and neck (HNC), prostate (PC), and cervical cancer (CC) we found 5890 cases of AD+CA or 2.15%. While lung represented the largest sample, colorectal (CRC) cancer was responsible for the largest proportion of concurrent AD+CA cases at 3.52% of all CRC. Black and Latino CRC, HNC patients had higher than overall prevalence of AD+CC. Black CRC patients had 6.13% AD+CA vs White 3.27 and Latino HNC patients reported 5.06% vs 3.25 White. Earlier stage patients had higher AD+CA vs later stages for CRC, HNC, and CC. The opposite was true for LC. Finally, women had slightly higher prevalence of AD+LC but significantly higher AD+CRC and AD+HNC compared to men.

